# Health-Related Quality of Life Before and After Use of a Smartphone App for Adolescents and Young Adults With Cancer: Pre-Post Interventional Study

**DOI:** 10.2196/13829

**Published:** 2019-10-03

**Authors:** Helle Pappot, Gry Assam Taarnhøj, Abbey Elsbernd, Maiken Hjerming, Signe Hanghøj, Marc Jensen, Kirsten Arntz Boisen

**Affiliations:** 1 Department of Oncology Rigshospitalet Copenhagen University Hospital Copenhagen Denmark; 2 University of Kansas School of Medicine Kansas City, KS United States; 3 Department of Hematology Rigshospitalet Copenhagen University Hospital Copenhagen Denmark; 4 Center of Adolescent Medicine Department of Pediatric and Adolescent Medicine, Rigshospitalet Copenhagen University Hospital Copenhagen Denmark

**Keywords:** adolescent, young adult, cancer, mHealth, smartphone, survivorship, quality of life

## Abstract

**Background:**

Adolescent and young adult (AYA) patients with cancer are a group with underexplored needs throughout treatment and in survivorship. This missing knowledge can influence their quality of life (QoL). Given this fact, we have developed a smartphone app based on a cocreation process and have an investigation of QoL among users planned as part of pilot testing this app. Future research is warranted to determine the effect of mobile health (mHealth) tools such as smartphone apps among the AYA cancer population.

**Objective:**

The aim of this study was to investigate the feasibility of a smartphone app among AYA patients with cancer in active treatment and posttreatment, in a pilot test by measuring health-related QoL before and after the use of the app.

**Methods:**

Participants were recruited via the youth support initiative and social organization for AYAs with cancer, Kræftværket, based at Rigshospitalet, University Hospital of Copenhagen, Denmark. Participants were evenly distributed in active treatment and posttreatment groups. After written informed consent, all participants were asked to use the app Kræftværket as they deemed appropriate over a 6-week period. The participants were asked to complete the 30-item European Organization for Research and Treatment of Cancer Quality of Life Questionnaire before and after the 6-week period. The collected QoL data were analyzed with *t* tests to determine differences between groups and from baseline.

**Results:**

In total, we enrolled 20 participants, 10 in active treatment and 10 posttreatment (median time after treatment was 4 months) group. Median age of the participants was 25 years. No differences in QoL were seen at baseline (*P*=.65). The posttreatment group experienced a significant increase in overall QoL after the 6-week period (global QoL: baseline 62.5, SD 22.3; after 6 weeks 80.8, SD 9.7; *P*=.04). For the group in active treatment, the QoL remained stable throughout the 6 weeks.

**Conclusions:**

This study shows the feasibility and possible effect on QoL associated with the use of an mHealth tool in AYA patients. mHealth support tools are warranted for this population.

## Introduction

### Background

Adolescents and young adults (AYAs) with cancer is a patient group with unique and often underexplored and unmet needs throughout treatment and in survivorship [1-3]. These patients face challenges specific to their age in physical, emotional, and social domains, which may have a detrimental impact on their health-related quality of life (HRQOL) [4-7]. Improving HRQOL in AYA patients with cancer and AYA cancer survivors is of both clinical and research interest in the disciplines of hematology and oncology, particularly through the development of youth-oriented interventions [8].

Smartphone apps and other mobile health (mHealth) interventions have been increasingly used for AYAs with cancer, as well as for AYAs with other acute or chronic disease [9-11]. Apps are attractive tools for AYA oncology and hematology interventions because of their inherent capability of fulfilling a wide number of tasks, including social networking, health tracking, health promotion, and information provision [12,13]. In addition, they are particularly useful for AYA patients, who are often technologically savvy and high users of smartphone technology [13,14]. However, there has been criticism regarding the design of these smartphone apps, based upon limited youth input in design and incomplete or inadequate evaluation of these apps [9,10,15,16].

Despite these limitations, smartphone apps and other mHealth interventions still demonstrate potential to serve as useful interventions for AYAs with cancer and AYA cancer survivors. On the basis of a cocreation process, our research team at Rigshospitalet, University Hospital of Copenhagen, Denmark, has developed a smartphone app, Kræftværket, to improve the QoL in AYAs with cancer and AYA cancer survivors [17,18]. The Kræftværket app, which is named after a youth support initiative and social organization for AYAs with cancer out of Rigshospitalet with the same name, has been designed with 3 primary features: (1) a symptom and activity diary, (2) a communication network between app users, and (3) an information database including video content. All features were selected and refined using the process of cocreation, in which AYA input was used to determine app content and design. The evaluation protocol for this app has been designed according to 2 evaluation stages: pilot testing and implementation testing [19]. The pilot testing consists of a qualitative and quantitative launch of the app to a small group of 20 AYA patients with cancer and cancer survivors, with quantitative evaluation using the 30-item European Organization for Research and Treatment of Cancer Quality of Life Questionnaire (EORTC QLQ-C30) instrument, and qualitative evaluation using focus group interviews and think-aloud testing [19]. Results of pilot testing will be used to modify and improve the app for launch of the app to a larger number of participants for implementation testing.

### Objectives

The aim of this study was to present the quantitative HRQOL data from the Kræftværket app pilot testing.

## Methods

### Overview

Detailed methods on the Kræftværket app development project are described in 2 separate papers [17,19]. As mentioned previously, the Kræftværket app was designed with 3 primary features: (1) a symptom and activity diary, (2) a communication network between app users, and (3) an information database including video content. The communication network allowed for nonmediated group-based communication between users as requested by the users during the cocreation process. The study plan was to evaluate the feasibility of the use of the Kræftværket app while measuring QoL in a pre-post study design.

### Participants and Recruitment

Kræftværket is a youth support initiative and social organization for AYA patients with cancer aged 15 to 29 years based at Rigshospitalet University Hospital of Copenhagen, Denmark [17]. Inclusion criteria for app pilot testing were AYAs aged 15 to 29 years with prior Kræftværket initiative contact and access to a smartphone and the internet, including cellular data or Wi-Fi. Participants were excluded if they were unable to read and write in Danish, and if they participated in the cocreation activities that determined app content and design [19]. For the pilot-testing phase, 20 previous or current Kræftværket users were recruited to pilot-test the app and provide qualitative and quantitative feedback. Recruitment was targeted to obtain 10 participants currently undergoing treatment for cancer, and 10 participants who had completed treatment for cancer. Participants were invited to participate in the study through invitation in the open Facebook group Kræftværket Rigshospitalet or while participating in other Kræftværket initiatives at Rigshospitalet. No individual patient was approached. The demographic data were provided by the AYAs themselves.

### Pilot Testing

After obtaining informed consent, participants provided baseline measurements of HRQOL using the validated EORTC QLQ-C30 instrument for overall and subdomain QoL measurement. No other EORTC modules or instruments were used throughout this pilot testing.

Participants were asked to download the Kræftværket app and contact the software developer, Daman, to obtain a log-in, followed by using the Kræftværket app over the course of 6 weeks. Upon completing written informed consent, participants were shown the app and the 3 features by youth coordinator MH, but they were not given any specific instructions on suggested frequency of app use; instead, they were asked to use the app as they deemed fit. At the end of the 6-week period, they were asked to complete a secondary EORTC QLQ-C30. The original protocol for app evaluation stated that participants would be prompted to complete the EORTC QLQ-C30 via the app; however, this instrument was completed in paper form because of changes in personal data protection rules.

At this time, additional qualitative data were collected by author SH in the form of semistructured focus group interviews and individual think-aloud tests. The qualitative data were collected after the completion of the QLQ-C30 after the 6-week period. Data from the qualitative aspects of this pilot test will be published in 2 separate papers [20,21].

### Statistical Analysis

Statistical analysis was performed with IBM SPSS version 25.0. Descriptive statistics and frequencies were used to display sociodemographic and clinical data. A paired *t* test was performed to determine differences in global QoL from baseline. Differences over time are illustrated by a boxplot. No statistical analyses were performed between groups as the sample size did not allow for between-group comparisons.

### Ethical Considerations

We obtained informed consent from all individual participants included in the study, whereas caregiver informed consent was given for patients younger than 18 years. All procedures performed in studies involving human participants were in accordance with the ethical standards of the institutional and national research committee and with the 1964 Helsinki declaration and its later amendments or comparable ethical standards. This research was exempt from review by an institutional review board or ethical authority under Danish law. Due to the Danish Data Protection law restrictions, the tracking data registered through the app of the patients participating in this study were not available. Permission to conduct the study was granted by the Data Protection Agency (j.nb. 2012-58-0004, i-suite nb.:6217).

## Results

### Participants and Recruitment

Over a 2-week period, 21 patients were approached at Kræftværket; of these, only 1 patient declined participation. In total, 20 recruited patients, 10 in active treatment and 10 posttreatment, completed written informed consent. Of these participants, 70% (14/20) were female. Among the participants in the active treatment group, the gender distribution was 60% (6/10) vs 40% (4/10) with a majority of females. This dispersion increased in the posttreatment group with 80% (8/10) women. Median age of the participants was 25 years, and for the posttreatment group, the median time elapsed from diagnosis to start of study was 4 months. The type of cancer was predominantly hematologic cancer (50%, 10/20) followed by breast cancer (20%, 4/20). Table 1 presents the demographic and clinical data.

### Pilot Testing

**Figure 1:**
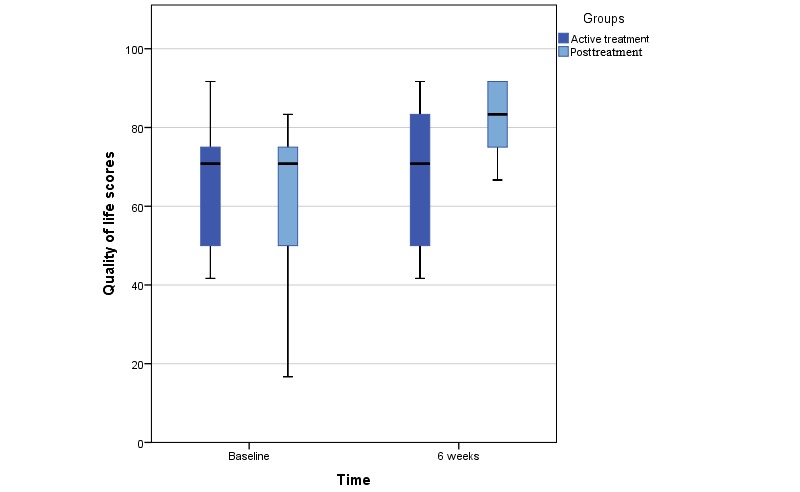
Boxplot of difference in baseline and 6-week global quality of life scores.

The mean global QoL at baseline was similar in the 2 treatment groups (active treatment group 66.67 vs posttreatment group: 62.5, *P*=.65); see boxplot in Figure 1. Table 2 shows the average EORTC QLQ-C30 domain scores. All domains are scored on a 0 to 100 scale. Although a higher global QoL score indicates better QoL, the symptom scales, for example, pain and nausea, are reversely scored, with a higher score indicating more impairment. These scores reveal a difference in baseline scores in favor of the posttreatment group for the subdomains role functioning, emotional functioning, cognitive functioning, and social functioning, and the single items for fatigue, nausea, dyspnea, insomnia, appetite loss, and financial difficulties. On the contrary, the single items on pain, constipation, and diarrhea were higher at baseline in the posttreatment group. Due to the large disparity between diagnoses and treatments received, no statistical analyses were performed for these data. A significant increase in global QoL was found for the posttreatment group from baseline to 6 weeks (difference estimate 18.3; 95% CI 1.5-35.1; *P*=.04). The paired analysis found no difference from baseline to 6 weeks after the use of the app for the group in active treatment (difference estimate 1.7; 95% CI –5.6 to 9.0; *P*=.61).

**Table 1:** Demographic and clinical information of participants (N=20).

Clinical data		All participants (N=20)	Active treatment group (n=10)	Posttreatment group (n=10)
**Gender, n (%)**				
	Male	6 (30)	4 (40)	2 (20)
	Female	14 (70)	6 (60)	8 (80)
Age (years), mean (range)		25 (16-29)	24 (19-29)	28 (16-29)
**Cancer type, n (%)**				
	Lymphoma	9 (45)	5 (56)	4 (44)
	Breast	4 (20)	3 (75)	1 (25)
	Head and neck	2 (10)	1 (50)	1 (50)
	Leukemia	1 (5)	0 (0)	1 (100)
	Testicular	1 (5)	0 (0)	1 (100)
	Ventricular	1 (5)	0 (0)	1 (100)
	Thyroid	1 (5)	1 (100)	0 (0)
	Brain	1 (5)	0 (0)	1 (100)
Median time posttreatment, months (range)		—^a^	—	4 (1-41)

^a^Not applicable.

**Table 2:** Mean value for the 30-item European Organization for Research and Treatment of Cancer Quality of Life Questionnaire scales at baseline and 6-week measurement.

EORTC QLQ-C30^a^ domains	Active treatment, mean (SD)		Posttreatment, mean (SD)	
	Baseline	6 weeks	Baseline	6 weeks

Global health status/quality of life	66.67 (17.12)	68.33 (17.48)	62.5 (22.31)	80.83 (9.66)
Physical functioning	74.67 (30.27)	77.33 (30.66)	80.67 (15.85)	87.33 (15.85)
Role functioning	55 (32.44)	60 (27.44)	68.33 (30.88)	81.67 (32.82)
Emotional functioning	65.83 (27.34)	63.33 (21.59)	68.67 (28.47)	82.67 (12.25)
Cognitive functioning	55 (36.89)	71.67 (33.38)	63.33 (33.15)	86.67 (17.21)
Social functioning	73.33 (30.63)	75 (25.15)	78.33 (30.48)	81.67 (21.44)
Fatigue	50 (21.11)	44.44 (23.42)	42.22 (22.71)	33.33 (13.86)
Nausea	20 (31.23)	23.33 (31.62)	10 (11.65)	11.67 (13.72)
Pain	22 (23.64)	25 (30.68)	33.33 (34.25)	13 (15.32)
Dyspnea	33.33 (27.22)	23.33 (31.62)	16.67 (17.57)	6.67 (14.05)
Insomnia	50 (28.33)	36.67 (39.91)	30 (33.15)	30 (29.19)
Appetite loss	30 (36.68)	26.67 (37.84)	10 (16.10)	6.67 (14.05)
Constipation	6.67 (14.05)	13.33 (32.20)	16.67 (32.39)	16.67 (23.57)
Diarrhea	10 (22.50)	13.33 (32.20)	13.33 (23.31)	6.67 (14.05)
Financial difficulties	50 (39.28)	50 (39.28)	13.33 (23.31)	3.33 (10.54)

^a^EORTC QLQ-C30: 30-item European Organization for Research and Treatment of Cancer Quality of Life Questionnaire.

## Discussion

### Principal Findings

This study has demonstrated feasibility and possible impact of a smartphone app on the HRQOL in this small AYA population. Our results show that QoL may be positively affected in a group of patients posttreatment after a short period of use of a specifically designed mHealth tool.

The field of mHealth technologies designed for individuals with cancer is in a period of rapid growth and development. The present smartphone app may fill the gap for a population in need of added support. Other mHealth tools may have the same potential but have until now lacked testing and validation in an appropriate population [9,10,15]. Several studies have affirmed the feasibility of mHealth use in different populations, thus creating opportunities for similar strategies in other cancer populations, such as older adults, in both active treatment and survivorship, as well as AYA with other conditions.

The increase seen in QoL may be explained by several factors. Posttreatment patients not in daily contact with fellow patients or hospital personnel may experience improvement in QoL using the Kræftværket app owing to added social interaction and increased sense of belonging. A study by Kaal et al introduces the relationship between empowerment and HRQOL in AYA patients and demonstrates that empowerment is positively associated with HRQOL [22]. Empowerment has previously been theorized as a broad construct of intrapersonal, interactional, and behavioral components [23]. When extending these constructs to our data, the interactional component (eg, the social interaction feature of the app) may be able to explain the development seen, although, in this study, we do not have data on which of the 3 individual elements of the app were used most frequently by participants. Thus, we cannot be sure if the increase in QoL is a result of increased empowerment through an element of social interaction. Patients still in active treatment have regular contact with their health care provider, and, in our case, the Kræftværket group and associated personnel. As such, they may not experience an added sense of belonging through using an interactive app such as Kræftværket. This may be an explanation for this group of participants not experiencing the same benefits as the posttreatment group. However, for some AYA populations, for example, AYAs with hematological cancers going through burdensome treatments, one may expect a decrease in QoL. In this study, 50% of the AYAs in active treatment were treated for hematological cancer, yet no decrease in QoL was seen for this group. Due to the study design, the course of QoL for patients not exposed to the app cannot be reported. This can only be explored in a randomized trial design. However, literature informs us of the many issues presumed to affect the QoL of these patients after treatment. Therefore, one would expect a decrease in QoL after treatment [24]. This decrease is not seen in this study, thus emphasizing the possible effect of the Kræftværket app.

The levels of global QoL recorded at baseline by EORTC QLQ-C30 are comparable with previous published data for posttreatment AYA patients [25,26], although lower than the levels in an adult cancer population [27]. The difference in age compared with the adult cancer populations may have an impact on QoL because younger people in important developmental phases of life have higher expectations to life and, thus, a worse experience of the misfortunes of life [28]. In addition, an explanation for the lower overall QoL could be the dominant group of hematologic cancer patients in this and similar AYA studies [25,26]. These patients go through a longer and by many accounts more demanding oncological treatment. Interestingly, the 6-week global QoL value for the posttreatment group in this study was comparable with an age-standardized group of German citizens without cancer as shown by Geue et al [25], thus demonstrating a potential for the Kræftværket app in this population.

Given the potential of the current app, and similar apps for an AYA population in need of added support, the access to smartphone technology is a requirement. A review from 2015 indicates a concern for individuals of lower sociodemographic status and restricted access to smartphones as high as 25% [10]. Restricted access would undoubtedly create a selection bias of whom would benefit from this technology. However, several surveys performed in 2015-2018 from comparable countries have shown very high access to smartphone for the age group of 12 to 34 years (89%-98%) [14,29,30].

Strengths of this study include the feasibility testing of a smartphone app for patients with cancer including the possible positive effects its use has on these patients. Moreover, this app has been tested in a diverse AYA population with a large variety of cancer diseases represented. In addition, the app is tested in 2 different settings, during active treatment and posttreatment, thus implying its use in a survivorship setting for the potential benefit of a large population. Finally, the cocreation process during the development of this app and during the process of this study with direct patient involvement (coauthor MJ) may certainly have a role in the success of its use, hence encouraging a similar process in the development of future apps [19].

Some limitations do need to be addressed. First, the pre-post study design in a very small population clearly represents a limitation and lessens the possible interpretations of the presented data. In addition, owing to the data protection restrictions in Denmark, the tracking information of app use was not available to report. Therefore, we do not know how and to what extent the participants used the app, thereby rendering it difficult to conclude why the posttreatment group experienced an increase in QoL, which may have been seen even without the use of the app. In addition, because the clinical information of cancer disease was self-reported by the patients, we did not have data on the stage of disease or specific oncological treatment. These data may have contributed to a more thorough explanation of the observed lower global QoL scores in these participants, as patients at the end of their lives would expect to experience a decrease in QoL [22]. Finally, although the EORTC QLQ-C30 has been used in more than a thousand clinical trials and daily clinics, several groups have allegated that this instrument is noncomprehensive and does not cover all the QoL aspects important to AYAs [4-6]. In previous studies by Nightingale et al and Quinn et al, 3 constructs were described as lacking for the AYA population: perceived sense of self, relationships, and parenthood [31,32]. Therefore, the QoL results may not portray the complete picture of these patients’ QoL.

### Perspectives

This study shows the feasibility and possible positive effects on QoL by use of an mHealth tool in AYA patients. The study gives hope to the use of mHealth tools to improve QoL in AYAs with cancer. Future studies evaluating the impact of the Kræftværket app in the AYA cancer population will show the effect implementation of mHealth tools may have on this patient group.

## Abbreviations

AYA: adolescent and young adult
EORTC QLQ-C30: 30-item European Organization for Research and Treatment of Cancer Quality of Life Questionnaire
HRQOL: health-related quality of life
mHealth: mobile health
QoL: quality of life
